# Family Resilience and Its Influencing Factors Among Patients With Lung Cancer Based on Double ABC‐X Theoretical Framework

**DOI:** 10.1002/cam4.70868

**Published:** 2025-04-15

**Authors:** Ziyi Shen, Yan Fang, Chengcheng Li, Xin Luo, Junling Cui, Yanchang Liu, Jingfang Hong

**Affiliations:** ^1^ School of Nursing Anhui Medical University Hefei Anhui China; ^2^ Anhui International Joint Research Center of Nursing Science Hefei Anhui China

**Keywords:** double ABC‐X model, family resilience, lung cancer patients, structural equation modeling

## Abstract

**Objective:**

The aim of this study was to evaluate the family resilience and its psychosocial influencing factors of patients with lung cancer. Relationships between variables and pathways were also explored based on the Double ABC‐X as the theoretical framework.

**Methods:**

A cross‐sectional survey of 318 lung cancer patients was conducted in a tertiary hospital in Anhui, China. The questionnaires included a general information survey, the Chinese perceived stress scale, the Connor‐Davidson Resilience Scale, the social support scale, the cognitive emotion regulation scale, the self‐efficacy scale, the medical coping modes questionnaire, and the family resilience scale. Data entry was performed using EpiData3.1 software, while data processing and analysis were conducted with SPSS 26.0. The structural equation modeling was fitted and validated using AMOS 26.0 software.

**Results:**

Family resilience levels were found to be moderately above average. Linear regression analysis indicated payment method, employment status, time since diagnosis, confrontative coping style, Positive Cognitive Emotion Regulation (PCER), perceived stress, social support, and personal resilience as the main influencing factors, which could explain 71.1% of the variation of family resilience in individuals with lung cancer. The final model comprises nine significant pathways. Cognitive emotion regulation, self‐efficacy, and social support all have direct positive effects on family resilience (*β* = 0.202, *β* = 0.272, *β* = 0.298). Personal resilience, social support, and self‐efficacy significantly positively influence confrontive coping modes (*β* = 0.293, *β* = 0.175, *β* = 0.121). Individual resilience has a significant positive effect on self‐efficacy, with a path coefficient of *β* = 0.377. Submissive coping modes are notably enhanced by perceived stress, as indicated by a path coefficient of *β* = 0.516, while personal resilience negatively affects submissive coping styles, as indicated by a path coefficient of *β* = −0.188.

**Conclusions:**

This study highlights the importance of psychosocial factors, such as cognitive emotion regulation, self‐efficacy, and social support, in enhancing family resilience in lung cancer patients. Clinically, interventions targeting these factors might significantly improve family functioning and coping mechanisms, contributing to better patient and family outcomes during cancer treatment and care.

## 1 Introduction

Lung cancer, a prominent global public health concern, severely impacts human life and health. According to the latest cancer data released by GLOBOCAN 2022, the number of new lung cancer cases and deaths were 2.4 million and 1.8 million respectively, both ranking first globally. Additionally, the occurrence and fatality rates of lung cancer in Asian countries far exceed those in European and American countries [1]. In China, the incidence and mortality rates of lung cancer remained high in 2022, with 810,000 new cases and 710,000 deaths, demonstrating a trend towards affecting younger populations [2].

With the advancement of medical technology, small pulmonary nodules can be detected during physical examinations. However, due to economic and other factors, the screening rate among high‐risk populations is relatively low. Consequently, most patients are diagnosed at an advanced stage, missing the opportunity for surgical intervention [3, 4]. A multicenter observational study in China demonstrated that among lung cancer patients with known staging at initial diagnosis, the proportion diagnosed with Stage III–Stage IV disease was as high as 67.4% (9455/14,024) [5]. It is noteworthy that due to the aggressive nature of advanced lung cancer and the complexities of its treatment, old age and comorbidities may have a more pronounced impact on lung cancer patients. The intensive treatments such as radiotherapy, chemotherapy, and immunotherapy, while extending survival, might lead to greater functional decline and affect the overall quality of life, especially in older patients or those with additional comorbidities. Additionally, the recurrent need for treatment not only inflicts significant physical distress but also leads to elevated levels of stigma, anxiety, and depressive symptoms [6], causing patients to become increasingly dependent on family members, who consequently bear a substantial caregiving burden. Throughout the prolonged caregiving process, the immense physical strain also gives rise to negative emotions among family members, and the ensuing psychological distress and anxiety can further adversely affect the patient's mental health and quality of life [7]. Research indicates that families can influence the health of their members through emotional responses, genetics, and environmental factors, prompting researchers to consider the family as a cohesive unit. This approach allows for the study of the positive forces exhibited by families in coping with illness. Consequently, the significance of the family in supporting lung cancer patients has gradually come to the attention of scholars [8].

Family resilience, rooted in positive psychology, sheds light on the strategies employed by families living in challenging environments to not only confront adversity but also to ensure the preservation of their system's integrity and functionality without impairment [9]. Scholars such as McCubbin have proposed the Double ABC‐X Model, based on Hill's ABC‐X Family Crisis Model, to explain the processes of coping and adaptation among family members when facing critical events [10]. In the context of health, the Double ABC‐X Model has been extensively applied to understand how families cope with health‐related crises and the long‐term effects of these crises. One of the key advantages of the Double ABC‐X Model is its ability to capture not only the initial family response to a crisis but also the long‐term adaptation processes, including how families reorganize resources, develop new coping mechanisms, and sustain their overall functioning. This holistic perspective provides deeper insights into family resilience and the preservation of family integrity in the face of ongoing health challenges [11].

A meta‐analysis on family resilience in cancer patients identified a total of 20 articles published in the relevant field, with the study populations primarily focusing on children with cancer, breast cancer patients, as well as their care‐providers. Overall, the level of family resilience in cancer patients has been found to range from moderate to high, with factors influencing these resilience levels primarily including demographic, disease‐related, psychological, and economic factors, among others [9, 12]. Research on family resilience in lung cancer patients remains limited. Existing studies suggest that the level of family resilience in this group is moderate, with key influencing factors identified as demographic variables (e.g., age, gender, and education), disease‐related factors (e.g., cancer stage and treatment type), personal resilience, and self‐efficacy. Specifically, higher levels of resilience are often associated with younger age, greater social support, and more adaptive coping mechanisms [7, 8]. However, most studies concentrate on specific populations, such as chemotherapy patients or elderly female patients [13, 14], often with restrictive inclusion criteria that limit generalizability. Furthermore, theoretical models to guide these studies are lacking. To address these gaps, this study aims to assess the levels of family resilience at different stages of lung cancer and to establish a hypothetical model of influencing pathways based on the Double ABC‐X model. The model will clarify the pathways and coefficients through which various factors affect family resilience in lung cancer patients.

### 1.1 Theoretical Basis

**FIGURE 1:**
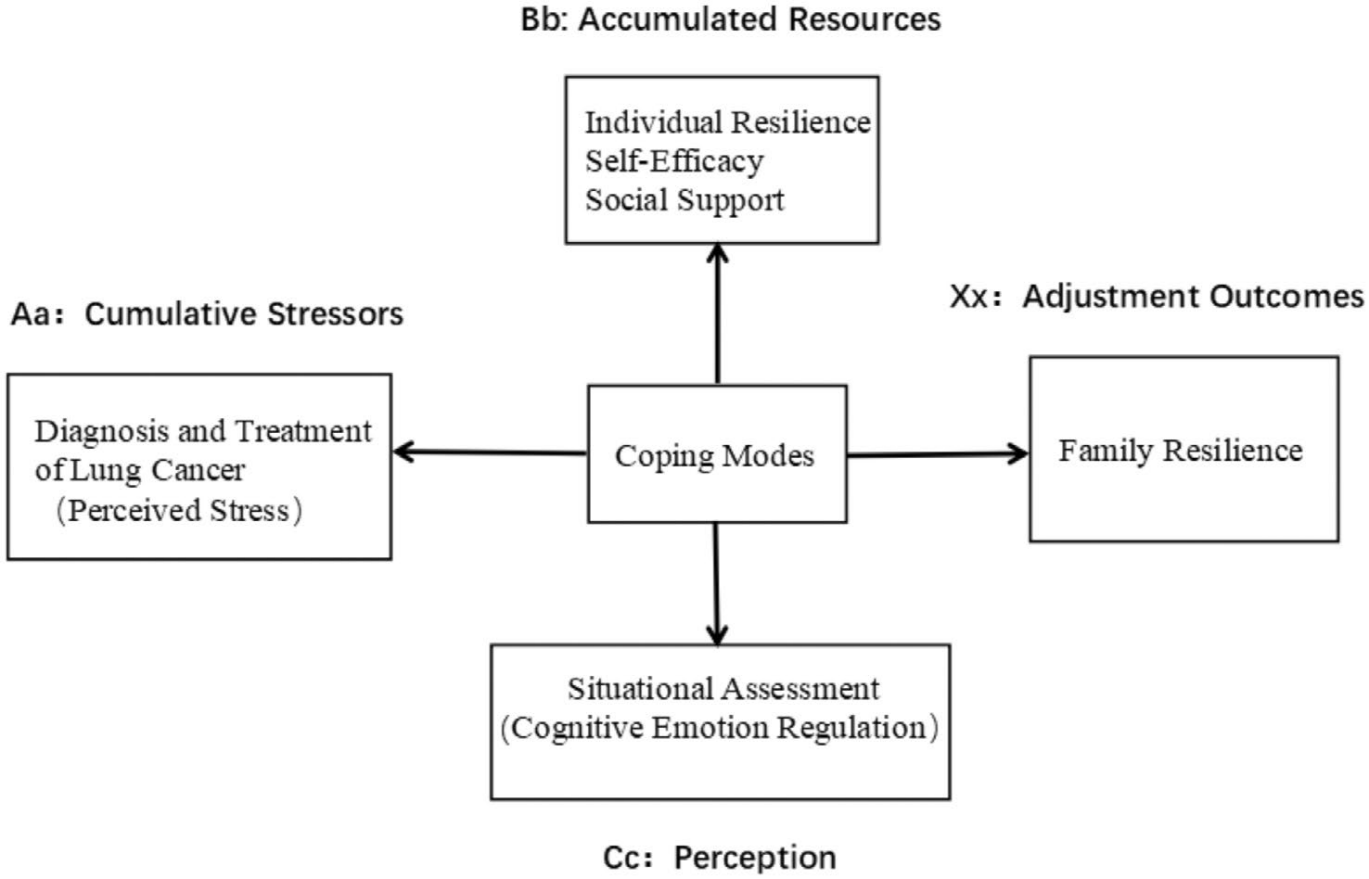
Theoretical model of factors influencing family resilience among lung cancer patients.

The Double ABC‐X Model comprises four elements: Aa, Bb, Cc, and Xx. Aa represents cumulative stress events, which may originate from the illness itself; Bb denotes the resources that a family can mobilize in the face of a crisis, primarily including personal capabilities, family resources, and social support; Cc signifies the family's perception of the crisis event and the resources obtained; Xx represents the family adaptation balance, which is the outcome of adjusting to and coping with the stressors [10]. In this study, the diagnosis of lung cancer among family members is considered a stressor event, meaning that the cumulative stress event of lung cancer diagnosis and treatment serves as the Aa factor, measured through the perception of stress by the patient. Self‐efficacy is the ability of lung cancer patients to execute a specific goal, while psychological adaptation is the dynamic and multidimensional process and outcome of coping with health threats. This study employs personal resilience, self‐efficacy, and social support as resources that can be mobilized by lung cancer patients and their family members in response to stress events, constituting the Bb factor. The Cc factor pertains to the cognitive appraisal of Xx, Aa, and Bb, evaluated through cognitive emotion regulation. Coping Modes act as the “intermediate steps” that guide the family's reaction to the stressor (lung cancer diagnosis and treatment), how resources are mobilized (personal resilience, social support), and how the family perceives and adjusts to the stressor over time. At last, the Xx factor denotes family resilience, which involves minimizing or alleviating the likelihood of the family system being compromised and reestablishing inherent stability, achieved through interaction among family members. (For details, see Figure 1).

## 2 Methods

### 2.1 Design and Participants

This research, of a cross‐sectional nature, was conducted at a tertiary, grade‐A comprehensive hospital in Hefei, Anhui Province. A convenience sampling approach was utilized to conduct a survey of lung cancer patients from January 2024 to June 2024 (No specific time point after surgery or chemotherapy was required for inclusion, as the aim was to capture patients at various stages of their treatment or recovery. Data were categorized by clinical stages and demographic factors to account for patient variability). The criteria for inclusion are specified as follows: (1) Individuals who have been diagnosed with lung cancer based on pathological examination; (2) Age ≥ 18 years, cohabitating with family members; (3) Awareness of their own condition and cancer diagnosis; (4) Possess certain comprehension and verbal expression abilities; (5) Volunteers for this study and sign an informed consent document. The following are the criteria for exclusion: (1) History or current presence of mental illness and consciousness disorders; (2) Concomitant with other malignant tumors or severe diseases; (3) Participation in other clinical trials. The sample size was calculated using a cross‐sectional survey based on the formula for mean and its confidence interval: *N* = 4U_*α*_^2^·*S* ^2^/*δ* ^2^, *α* = 0.05, U_*α*_ = 1.96. Referring to analogous research findings: the standard deviation *S* = 33.54, and the permissible error is taken as 0.25 times the standard deviation [8], hence *δ* = 8.385. Substituting these values into the formula yields *N* = 4*1.96^2^*33.54^2^/8.385^2^ ≈ 246. Considering a 20% non‐response rate, at least 308 cases need to be included. A total of 329 questionnaires were distributed in this study. After excluding 11 incomplete responses, 318 valid questionnaires were returned, resulting in an effective response rate of 96.7%.

### 2.2 Data Collection

This study was sanctioned by the Ethics Committee of Anhui Medical University (approval number 83242303). Data collection was carried out by researchers who had obtained their nursing qualifications. During the survey, researchers thoroughly informed patients and their families about the objective and relevance of this research. To ensure patient privacy, all data gathered in this study was maintained absolutely confidential and used only for this research project. After obtaining the consent of the patients, an informed consent form was signed. The questionnaires were distributed by the researchers themselves and collected on‐site, with each questionnaire being checked individually. Questionnaires with missing content that could not be completed were considered invalid. For patients with low educational levels, poor vision, or difficulty understanding the questionnaire items, researchers used uniform guideline language to explain and conducted a question‐and‐answer survey while recording the responses truthfully to ensure the quality of the questionnaires.

### 2.3 Variables and Instruments

The detailed information regarding the scales used can be found in Table S1.

1. General information questionnaire

The patient's demographic information included gender, age, education level, religious beliefs, monthly family income, work status, etc. The patient's disease‐related information was obtained from cancer staging, treatment methods, presence of metastasis, etc.

2 Family resilience assessment scale (FRAS)

The Family Resilience Assessment Scale was compiled by Chinese scholar Dai Yan [15]. The scale comprises 10 first‐order dimensions and 2 second‐order dimensions. The first‐order dimensions include distress interpretation, positive prospecting, problem‐solving, life excellence, emotional sharing, intimate harmony, planned life, social support, clear communication, and cooperative coordination. The two second‐order dimensions are family beliefs and family strengths. The scale employs a Likert‐5 rating system, with item 30 being reverse‐scored. Scores range from 49 to 245, with higher values suggesting increased family resilience. The scale demonstrates good internal consistency, as evidenced by a Cronbach's alpha coefficient of 0.912 [16].

3 Chinese perceived stress scale (CPSS)

The Perceived Stress Scale was originally compiled by Cohen and his colleagues in 1983 [17], and later translated and revised by Yang [18]. The scale comprises 14 items, split into two categories: tension and a sense of loss of control, with the latter being reverse‐scored. This scale uses a Likert‐5 rating method, where scores from 0 to 4 correspond to “never”—“always.” Increased scores suggest heightened levels of perceived stress in patients. This scale has been widely applied among cancer and chronic disease patients in China, with a Cronbach's alpha coefficient of 0.78 [19].

4 Cognitive emotion regulation questionnaire (CERQ)

The Cognitive emotion regulation questionnaire was first developed by Garnifsk and colleagues [20]. It was later translated and revised by Zhu et al. The scale consists of 36 items, including both Positive Cognitive Emotion Regulation (PCER) and Negative Cognitive Emotion Regulation (NCER), which can be further divided into 9 subcategories. A Likert 5‐point scoring system is used, with “Never,” “Almost never,” “Sometimes,” “Almost always,” and “Always” corresponding to scores of 1–5, respectively. Higher scores indicate that the participants are more likely to adopt such cognitive coping strategies when facing negative events. The Cronbach's alpha coefficient is 0.81 [21].

5 Medical coping modes questionnaire (MCMQ)

The Medical Coping Modes Questionnaire was first developed by Feifel et al. [22], which was later translated and revised by Shen. The scale consists of 20 items, divided into three dimensions: confrontation, resignation, and avoidance. A Likert 4‐point scoring method is used, with higher dimension scores indicating a greater tendency for individuals to adopt that particular coping strategy. The Cronbach's *α* coefficients for the three dimensions are 0.69, 0.60, and 0.76, respectively [23]. However, due to the low reliability score (0.393) of the avoidance dimension in this study, this dimension was not included in the analysis. This low score indicated insufficient internal consistency, affecting its reliability. Excluding this dimension was necessary. This approach is consistent with previous studies that have also excluded unreliable dimensions to improve result robustness [24, 25].

6 Connor davidson resilience scale (CD‐RISC)

The Connor‐Davidson Resilience Scale was compiled by Connor [26] which comprises 25 items and utilizes a Likert 5‐point scoring method, with higher scores reflecting stronger levels of resilience. The Chinese version of this scale was translated and adjusted by Zhang et al. It consists of 25 items, encompassing three dimensions: “Strength,” “Resilience,” and “Optimism.” The scale also employs a Likert 5‐point scoring method, with items spanning from “Strongly Disagree” to “Strongly Agree.” The overall score lies within the range of 0 to 100, with higher scores indicating greater levels of personal resilience. This scale has been validated among populations with diabetes, the elderly, and cancer patients in China, as revealed by a Cronbach's alpha coefficient of 0.910 [27].

7 General self‐efficacy scale (GSES)

The General Self Efficacy Scale was developed by German clinical and health psychologist Ralf Schwarze et al. The scale employs a Likert 4‐point scoring method and consists of 10 items; items are rated on a scale from “Strongly Disagree” to “Strongly Agree.” Greater scores suggest an elevated sense of self‐efficacy. The scale has a Cronbach's *α* of 0.834, demonstrating good internal consistency. The Chinese adaptation of the scale was conducted by Yang et al. and has been widely used in China with Cronbach's alpha coefficient of 0.87 [28].

8 Perceived social support scale (PSSS)

The Perceived Social Support Scale was compiled by Zimet [29] which comprises three dimensions and 12 items, measuring the perceived support from family, friends, and others. It employs a Likert 7‐point scoring method, ranging from “Strongly Disagree” to “Strongly Agree,” with composite scores ranging from 12 to 84; elevated scores indicate an increased degree of social support received by patients. The scale was translated into Chinese by Jiang, as indicated by a Cronbach's alpha coefficient of 0.899 [30].

### 2.4 Data Analysis

All questionnaire data were entered and verified by two individuals using EpiData3.1 software, and statistical analyses were performed with the aid of SPSS version 26.0. Frequency and constituent ratios were used to statistically describe the general demographic information of lung cancer patients. Normality tests showed that the data in this study conformed to normality, so they were described using mean ± standard deviation (Mean ± SD). Independent sample t‐tests and ANOVA tests were used to compare the differences in family resilience levels among lung cancer patients with different demographic characteristics; correlation analysis was conducted by employing Pearson correlation analysis, and multivariate analysis was performed using multiple linear regression analysis. Based on the above analyses, a structural equation model for family resilience in lung cancer patients was constructed using AMOS 26.0 software, and the path relationships between variables were explored. SEM was chosen because it allows for the examination of complex, multi‐variable relationships and provides a more comprehensive analysis compared to traditional methods like regression analysis, especially when latent variables and measurement errors are involved. The model fit was evaluated using multiple indices, including *χ* ^2^/df, Tucker‐Lewis Index (TLI), Comparative Fit Index (CFI), Root Mean Square Error of Approximation (RMSEA), and Normed Fit Index (NFI) for evaluation. Missing data were handled using Full Information Maximum Likelihood (FIML). All tests used two‐sided testing, and *p* < 0.05 suggests that the observed difference is statistically significant.

## 3 Result

### 3.1 Patient Demographic Characteristics

This study enrolled a total of 318 lung cancer patients, predominantly male (75.8%), with an age range of 31 to 86 years and a mean age of 65.25 ± 9.59 years, with family sizes of three members or fewer constituting 57.5% of the sample. The monthly income for 155 patients (48.7%) ranged from 3000 to 5000 Chinese RMB. Although the majority of patients were married (91.2%), their primary caregivers were their children (53.8%). The majority of patients (70.1%) were diagnosed within the last 6 months, with 58.2% in stage IV. Additionally, 38.7% had concurrent chronic diseases, and 59.4% had metastasis. Detailed demographic information of the lung cancer patients is presented in Table 1.

**TABLE 1:** The general information of lung cancer patients (*N* = 318).

Variables	Group	Frequency (*f*)	Percentage (%)
Gender	Males	241	75.8
	Female	77	24.2
Age (year)	< 60	87	27.4
M ± SD	60–75	182	57.2
65.25 ± 9.59	> 75	49	15.4
Household size	≤ 3	183	57.5
	4–6	123	38.7
	> 6	12	3.8
Monthly family income (CNY)	< 3000	81	25.5
	3000–5000	155	48.7
	5001–8000	64	20.1
	> 8000	18	5.7
Primary caregiver	Spouse	116	36.5
	Children	171	53.8
	Others	31	9.7
Education level	Primary and below	182	57.2
	Junior high school	89	28
	High school/Secondary school	32	10.1
	College and above	15	4.7
Payment method of medical expenses	Self‐funded	3	0.9
	Employee medical insurance	62	19.5
	Urban resident medical insurance	253	79.6
Religious beliefs	No	299	94
	Yes	19	6
Marital status	Unmarried	3	0.9
	Married	290	91.2
	Widowed	21	6.6
	Divorced	4	1.3
Work status	Incumbency	33	10.4
	Retirement	87	27.4
	Jobless	198	62.3
Cancer stage	I	9	2.8
	II	26	8.2
	III	98	30.8
	IV	185	58.2
Time since diagnosis	< 6 months	223	70.1
	6–12 months	35	11
	> 12 months	60	18.9
Treatment method	Surgery	21	6.6
	Radiation therapy	7	2.2
	Chemotherapy	133	41.8
	Immunotherapy	21	6.6
	Surgery + Chemotherapy	18	5.7
	Chemotherapy + Radiation therapy	6	1.9
	Radiation therapy + Immunotherapy	3	0.9
	Chemotherapy + Immunotherapy	109	34.3
Chronic diseases	No	195	61.3
	Yes	123	38.7
Metastasis	No	129	40.6
	Yes	189	59.4

### 3.2 Current Status of Family Resilience Among Patients

The overall mean score for family resilience among patients was (181.15 ± 21.61), indicating an above‐average level. The specific scores for each dimension are presented in Table 2.

**TABLE 2:** The total score and subscale scores of family resilience among lung cancer patients (*N* = 318).

Variables	Item count	Score range	Mean ± SD
Total score	49	91–232	181.15 ± 21.61
*Second‐order dimension*			
Family beliefs	17	37–82	61.40 ± 7.95
Family strengths	32	53–152	119.71 ± 14.26
*First‐order dimension*			
Distress interpretation	7	15–35	26.56 ± 3.97
Positive prospecting	6	14–30	21.76 ± 2.58
Problem‐solving	6	9–29	23.19 ± 2.90
Life excellence	4	6–19	13.08 ± 2.73
Intimate harmony	4	7–20	15.74 ± 2.33
Social support	4	4–20	11.72 ± 3.33
Planned life	3	5–15	10.71 ± 1.86
Emotional sharing	4	7–20	14.79 ± 2.65
Clear communication	5	8–25	20.08 ± 2.20
Cooperative coordination	6	9–30	23.47 ± 3.35

### 3.3 Analysis of Factors Influencing Family Resilience Among Patients

#### 3.3.1 Univariate Analysis of Demographic and Disease Factor

Patient demographic and disease data were employed as the independent variable. The results indicate that there are statistically significant differences in family resilience levels based on monthly household income, educational level, method of medical expense payment, religious beliefs, employment status, duration since diagnosis, and treatment modality. Details are presented in Table 3.

**TABLE 3:** Univariate analysis of demographic factors and family resilience in lung cancer patients.

Variables	Total score of family resilience			Family beliefs			Family strengths		
	Mean ± SD	T/F	*p*	Mean ± SD	T/F	*p*	Mean ± SD	T/F	*p*
*Gender*		−1.259	0.209		−1.625	0.105		−0.809	0.419
Males	180.29 ± 22.09			60.99 ± 8.10			119.35 ± 14.69		
Female	183.84 ± 20.00			62.68 ± 7.40			120.86 ± 12.90		
*Age (Year)*		1.812	0.165		2.652	0.072		1.647	0.194
< 60	177.41 ± 22.92			59.78 ± 8.48			117.36 ± 14.71		
60–75	182.42 ± 21.35			61.85 ± 7.72			120.64 ± 14.39		
> 75	183.04 ± 19.71			62.57 ± 7.59			120.47 ± 12.66		
*Household size*		0.14	0.869		0.248	0.781		0.168	0.846
≤ 3	181.63 ± 20.30			61.61 ± 7.74			120.09 ± 13.24		
4–6	180.34 ± 23.04			61.02 ± 8.11			119.13 ± 15.40		
> 6	182.00 ± 27.27			62.08 ± 10.04			119.92 ± 14.26		
*Monthly family income (CNY)*		11.791	0.00***		11.786	0.00***		10.819	0.00***
< 3000	171.79 ± 21.87			57.95 ± 7.85			113.84 ± 14.66		
3000–5000	181.80 ± 20.84			61.60 ± 7.95			120.07 ± 13.36		
5001–8000	186.19 ± 19.83			63.38 ± 6.71			122.98 ± 13.97		
> 8000	199.72 ± 13.70			68.11 ± 5.54			131.61 ± 9.23		
*Primary caregiver*		2.988	0.052		3.1	0.046*		3.119	0.046*
Spouse	182.82 ± 21.48			62.52 ± 7.88			120.37 ± 14.33		
Children	181.61 ± 22.20			61.13 ± 8.20			120.36 ± 14.44		
Others	172.35 ± 16.84			58.68 ± 6.05			113.68 ± 14.26		
*Education level*		4.412	0.005**		4.044	0.008**		4.436	0.005**
Primary and below	179.73 ± 21.23			61.03 ± 7.74			118.59 ± 13.97		
Junior high school	180.67 ± 22.06			61.19 ± 7.93			119.48 ± 14.86		
High school/Secondary school	181.50 ± 21.47			60.84 ± 8.48			120.94 ± 13.75		
College and above	200.47 ± 15.67			68.27 ± 7.10			132.20 ± 9.34		
*Payment method of medical expenses*		3.543	0.03*		3.973	0.02*		3.302	0.038*
Self‐funded	185.45 ± 20.80			63.35 ± 7.56			122.26 ± 14.10		
Employee medical insurance	204.67 ± 6.11			69.00 ± 1.73			135.67 ± 4.93		
Urban resident medical insurance	179.81 ± 21.67			60.83 ± 7.98			118.91 ± 14.22		
*Religious beliefs*		−2.609	0.01*		−3.306	0.001**		−1.722	0.086
Yes	180.36 ± 21.58			61.03 ± 7.96			119.37 ± 14.34		
No	193.5 ± 18.55			67.16 ± 5.34			125.16 ± 12.00		
*Marital status*		2.005	0.113		0.687	0.561		2.829	0.039*
Unmarried	181.33 ± 24.19			61.33 ± 10.97			120.00 ± 13.23		
Married	181.82 ± 20.94			61.54 ± 7.84			120.25 ± 13.68		
Widowed	170.38 ± 27.27			59.10 ± 8.51			111.29 ± 19.29		
Divorced	188.50 ± 29.42			63.25 ± 12.69			125.25 ± 16.74		
*Work status*		3.309	0.038*		4.461	0.012*		2.969	0.053
Incumbency	180.94 ± 22.45			60.36 ± 8.29			120.58 ± 14.86		
Retirement	186.10 ± 22.19			63.54 ± 8.25			122.69 ± 14.66		
Jobless	179.01 ± 20.96			60.63 ± 7.62			118.28 ± 13.84		
*Cancer stage*		1.282	0.281		0.859	0.463		1.518	0.21
I	178.33 ± 12.46			60.11 ± 4.68			118.22 ± 8.79		
II	185.46 ± 22.05			61.96 ± 8.25			123.50 ± 14.19		
III	178.00 ± 24.95			60.42 ± 9.01			117.58 ± 16.50		
IV	182.35 ± 19.85			61.90 ± 7.40			120.39 ± 13.07		
*Time since diagnosis*		4.396	0.013*		5.033	0.007**		3.557	0.03*
< 6 months	183.31 ± 22.26			62.20 ± 8.07			121.02 ± 14.65		
6–12 months	172.97 ± 21.28			57.91 ± 8.03			115.06 ± 13.82		
> 12 months	177.88 ± 17.78			60.45 ± 6.89			117.53 ± 12.25		
*Treatment method*		2.135	0.04*		2.056	0.048*		2.094	0.044*
Surgery	180.62 ± 21.13			61.24 ± 7.13			119.38 ± 14.52		
Radiation therapy	180.14 ± 17.64			60.86 ± 6.72			119.29 ± 11.66		
Chemotherapy	182.45 ± 19.76			61.71 ± 7.27			120.77 ± 13.29		
Immunotherapy	189.90 ± 20.46			64.57 ± 7.28			125.33 ± 13.91		
Surgery + Chemotherapy	170.50 ± 28.81			57.22 ± 10.21			113.27 ± 19.48		
Chemotherapy + Radiation therapy	180.33 ± 38.65			60.00 ± 15.30			120.33 ± 23.59		
Radiation therapy + Immunotherapy	209.67 ± 6.02			71.67 ± 2.52			138.00 ± 5.20		
Chemotherapy + Immunotherapy	179.06 ± 21.28			60.95 ± 7.99			117.94 ± 13.62		
*Chronic diseases*		−0.068	0.946		−0.626	0.532		0.175	0.175
No	181.08 ± 23.46			61.17 ± 8.48			119.82 ± 15.50		
Yes	181.25 ± 18.40			61.75 ± 7.07			119.54 ± 12.10		
*Metastasis*		0.047*	0.962		−0.002**	0.999		0.112	0.911
No	181.22 ± 23.40			61.40 ± 8.42			119.82 ± 15.58		
Yes	181.10 ± 20.37			61.40 ± 7.65			119.64 ± 13.32		

* *p* < 0.05.

** *p* < 0.01.

*** *p* < 0.001.

#### 3.3.2 Correlations Between Psychosocial Variables and Family Resilience

The total score for patient perceived stress and its various dimensions exhibited a negative correlation with the overall family resilience score. The dimension of surrender in medical coping showed a negative correlation with both the total score and individual dimensions of family resilience. In contrast, social support, self‐efficacy, personal resilience, PCER, and the confrontational coping modes showed positive correlations with family resilience. Detailed correlation values are available in Table 4.

**TABLE 4:** Descriptive analysis and correlation analysis between patients' perceived stress, social support, self‐efficacy, personal resilience, cognitive emotional regulation strategies, coping styles, and family resilience.

Variables	Mean ± SD	Family beliefs	Family strengths	Total score of family resilience
Total score of stress perception	22.85 ± 7.06	−0.072	−0.014	−0.147**
Sense of anxiety	11.47 ± 3.57	−0.056	−0.001	−0.153**
Sense of loss of control	11.37 ± 3.59	−0.086	−0.028	−0.136*
Total score of social support	67.94 ± 9.06	0.205**	0.408**	0.766**
Family support	23.86 ± 2.82	0.185**	0.411**	0.743**
Friends support	21.99 ± 3.45	0.209**	0.373**	0.716**
Others support	22.09 ± 3.26	0.188**	0.384**	0.729**
Total score of self‐efficacy	24.69 ± 5.16	0.296**	0.313**	0.208**
Total score of individual resilience	65.83 ± 9.72	0.302**	0.299**	0.653**
Resilience	35.01 ± 5.29	0.257**	0.265**	0.639**
Strength	22.00 ± 3.81	0.225**	0.243**	0.602**
Optimism	8.82 ± 2.03	0.354**	0.287**	0.337**
Cognitive emotion regulation				
PCER	68.67 ± 6.07	0.314**	0.306**	0.403**
NCER	62.13 ± 11.30	0.008	0.104	0.178**
Medical coping				
Confrontation	17.61 ± 4.32	0.380**	0.394**	0.396**
Surrender	8.65 ± 2.69	−0.269**	−0.288**	−0.288**

* *p* < 0.05.

** *p* < 0.01.

### 3.4 Multiple Regression Analyses

The score of family resilience for patients was designated as the dependent variable, while variables demonstrating statistical significance from both univariate and correlation analyses were chosen as independent variables. A multiple linear regression model was constructed. The regression analysis results showed that payment method, employment status, duration since diagnosis, confrontational coping modes, PCER, stress perception, social support, and personal resilience were the main influencing factors on the level of family resilience among lung cancer patients (*p* < 0.05), collectively explaining 71.1% of the variance. Details are presented in Table 5.

**TABLE 5:** Results of multiple linear regression analysis on factors influencing family resilience in lung cancer patients.

Variables	Unstandardized coefficients	Standard error	Standardized coefficients	*t*	*p*	VIF
	*B*		Beta			
(constant)	38.415	11.355		3.383	0.001	
Payment method of medical expenses	−6.157	1.891	−0.123	−3.256	0.001	1.575
Work status	2.283	1.161	0.072	1.967	0.05	1.451
Time since diagnosis	−3.568	0.893	−0.131	−3.997	0.00	1.178
Confrontation	0.413	0.182	0.08	2.268	0.024	1.458
PCER	0.32	0.141	0.09	2.264	0.024	1.731
Stress perception	0.243	0.123	0.08	1.982	0.048	1.759
Individual resilience	0.714	0.1	0.32	7.118	0.00	2.235
Social support	1.289	0.091	0.54	14.132	0.00	1.605
*F*	53.030**					
*R* ^2^ (Adjusted *R* ^2^)	0.722 (0.711)					

*Note:* ** indicates that the model is highly significant (*P* < 0.01).

### 3.5 Structural Equation Model Analysis of Factors Influencing Family Resilience Among Lung Cancer Patients

#### 3.5.1 Based on the Double ABC‐X Theoretical Framework, a Hypothetical Model Was Constructed

**FIGURE 2:**
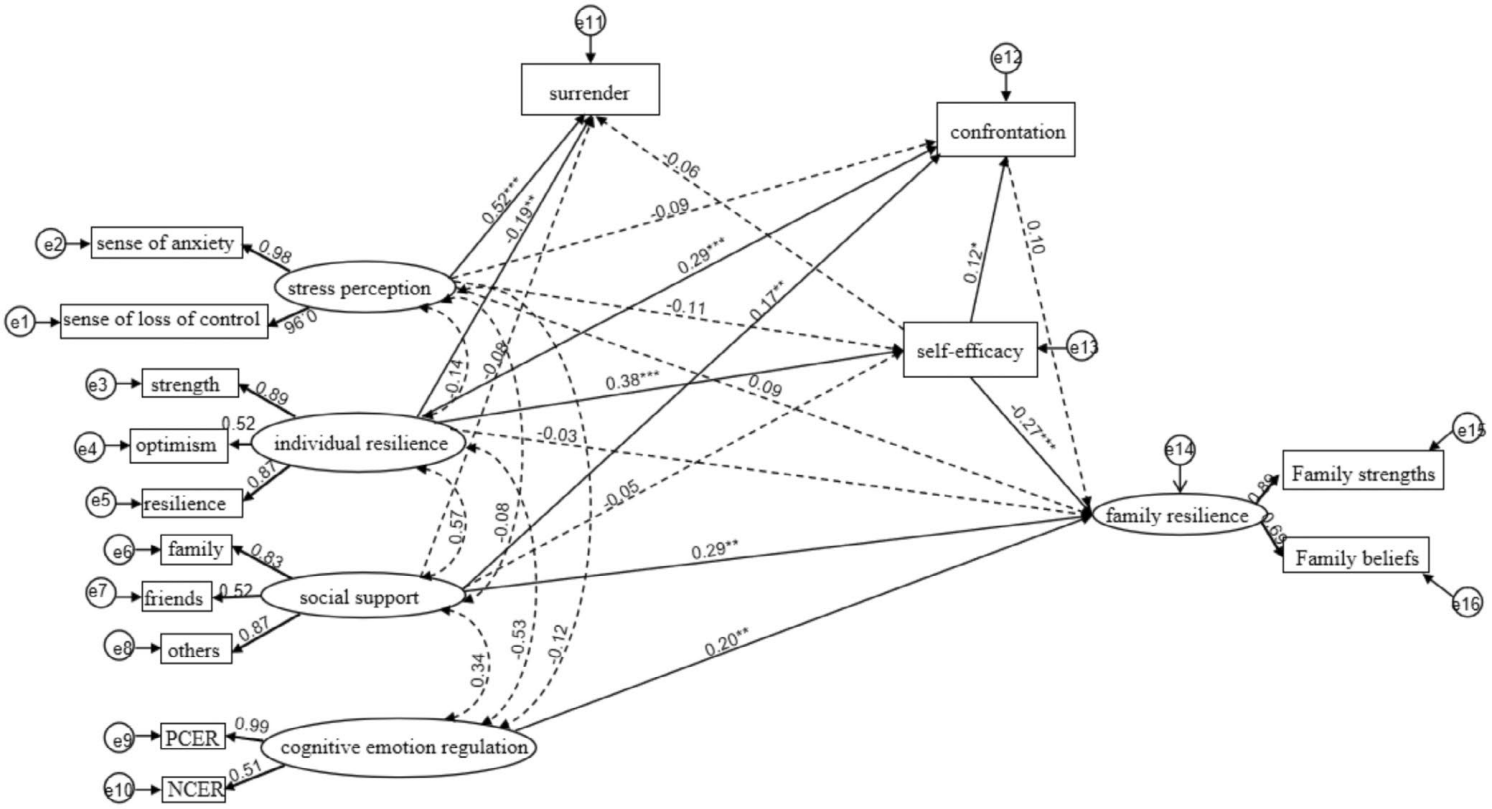
Structural equation model of factors influencing family resilience in lung cancer patients. *< 0.05, **< 0.01, ***< 0.001.

To verify whether the collected data could effectively elucidate the hypothetical model proposed in this study, a goodness‐of‐fit test was conducted on the model. The results showed: *χ* ^2^/df = 2.919, CFI = 0.957, TLI = 0.935, NFI = 0.937, RMSEA = 0.078, indicating satisfactory fit. Details of the model are presented in Figure 2.

#### 3.5.2 The Structural Equation Model Revealed Nine Significant Pathways

Cognitive emotional regulation, self‐efficacy, and social support had direct positive effects on family resilience. Personal resilience, social support, and self‐efficacy significantly influenced confrontational coping modes. Additionally, individual resilience positively affected self‐efficacy, while stress perception positively influenced surrender, which was negatively impacted by individual resilience. These path coefficients are summarized in Table 6.

**TABLE 6:** Results of path analysis for the model of factors influencing family resilience in lung cancer patients.

Path relationship	*β*	SE	CR	*p*
Self‐efficacy ← Individual resilience	0.377	0.122	4.722	0.00
Confrontation ← Individual resilience	0.293	0.101	3.721	0.00
Confrontation ← Social support	0.175	0.084	2.717	0.007
Confrontation ← Self‐efficacy	0.121	0.045	2.239	0.025
Family resilience ← Cognitive emotion regulation	0.202	0.219	2.882	0.004
Family resilience ← Self‐efficacy	0.272	0.211	4.531	0.00
Family resilience ← Social support	0.298	0.393	4.039	0.00
Surrender ← Stress perception	0.516	0.039	10.303	0.00
Surrender ← Individual resilience	−0.188	0.054	−2.755	0.006

*Note:* *β*, standardized coefficients; CR, critical ratio; *p*, significance; SE, standard error of *B*.

## 4 Discussion

This study enrolled a total of 318 lung cancer patients, utilizing the Family Resilience Scale to assess their levels. The results indicated that the overall score for family resilience among lung cancer patients was 181.15 ± 21.61, with subscale scores for family belief and family strength being 61.4 ± 7.95 and 119.71 ± 14.26, respectively. This suggests that the level of family resilience among lung cancer patients is moderately above average, which is consistent with the findings of Yang and Lu's research [8, 14]. The survey found that among the 10 subdivided dimensions, lung cancer patients scored lower in the dimensions of “orderliness” and “social support,” with scores generally lower than those of other cancer patients [31, 32]. This indicates that there is still significant room for improvement in the level of family resilience among lung cancer patients.

The standardized regression coefficients reveal that personal resilience, social support, duration since diagnosis, and medical insurance payment method have the greatest impact on the level of family resilience among lung cancer patients. Resilient individuals are better equipped to adapt to and recover from stress. This in turn can foster adaptive coping strategies and improve their family resilience. These findings are consistent with Cui, who highlighted that individual resilience contributes to stronger family resilience in response to a shared stressor [32]. This study further confirms that lung cancer patients with higher personal resilience tend to experience greater family resilience, reinforcing the critical connection between individual and family resilience. Furthermore, social support has been validated in this study as a key factor in promoting family resilience. This aligns with the work of Wang [33], social support helps patients develop family cohesion, which in turn enhances overall family resilience. Additionally, the type of medical insurance payment, particularly the use of employee health insurance, was closely associated with higher family resilience. This finding indicates that medical insurance can alleviate financial burdens, allowing patients to focus on emotional and familial issues, thereby enhancing overall family resilience. It is noteworthy that there is currently a debate regarding the impact of the duration since diagnosis on the level of family resilience in lung cancer patients. Lin's study concluded that lung cancer patients diagnosed within 6–12 months possess a higher level of family resilience [34], whereas Chen believes that patients diagnosed for approximately 1 year exhibit the highest level of family resilience [35]. However, different studies have consistently shown that the level of family resilience in lung cancer patients changes dynamically over the course of the disease [36]. Therefore, subsequent large‐scale longitudinal tracking surveys should be conducted to better explore the impact of different time points on the family resilience of lung cancer patients.

In accordance with the Double ABC‐X theoretical model, this study constructed a structural equation model to analyze factors influencing family resilience among lung cancer patients. The results indicated that cognitive emotional regulation has a significant positive impact on the degree of family resilience among these patients. To our knowledge, this is the first article to explore the correlation between patient cognitive emotional regulation and family resilience. Existing research indicates that certain positive emotions and coping regulation abilities can enhance understanding and communication among family members, increase family cohesion, and thereby improve the level of family resilience in cancer patients [36]. This necessitates that future healthcare professionals implement targeted interventions. For instance, interventions such as cognitive‐behavioral therapy (CBT) and emotion‐focused family therapy (EFFT) can help patients and their families develop better emotional regulation strategies, improve coping mechanisms, and strengthen familial bonds, ultimately facilitating their attainment of an elevated level of family resilience.

This study found that self‐efficacy and social support also have a significant positive effect on the level of family resilience in lung cancer patients, which is consistent with the research results of Lu and Ke et al. [8, 36, 37]. This is primarily because patients with higher levels of self‐efficacy tend to proactively explore their own potential and actively seek solutions to mitigate the negative impacts of the disease, thereby promoting harmonious interactions among family members and achieving a higher level of family resilience. Research indicates that social support is an important external factor for cancer patients to positively face crises. Adequate external support can help patients better integrate into society and enhance their sense of well‐being [38]. Those lung cancer patients who have more social support often have a higher capacity to cope with stressful events, and their level of family resilience is also significantly higher than that of patients with low social support. Therefore, nurses should pay close attention in their clinical work to patients with low levels of social support who are unable to actively face the cancer crisis. They should assist patients in expanding their social support networks and encourage active coping with the disease, thereby enhancing the level of family resilience in individuals with lung cancer.

### 4.1 Study Limitations

This study represents a cross‐sectional investigation carried out at a single time point, taking relevant data from lung cancer patients at a certain point in time. Therefore, the causal relationship needs to be explored cautiously, and longitudinal studies still need to be conducted in the future to deeply explore the interconnections between various variables. In addition, the use of a convenience sampling method in this study presents a limitation, particularly in terms of generalizability. Since participants were selected from a single hospital, the findings may not be applicable to lung cancer patients from different regions, healthcare settings, or cultural contexts. This limits the ability to generalize the results to the broader lung cancer population. Future studies using random or stratified sampling across multiple centers are needed to enhance the external validity of the findings. Finally, the research method of this study was a questionnaire survey, which was more subjective. In the future, some objective indicators can be added to enrich the research methods.

### 4.2 Clinical Implications

This study constructed and validated a hypothetical model of the pathways affecting family resilience levels in lung cancer patients based on the Double ABC‐X model, assessed the level of family resilience in lung cancer patients, identified its related influencing factors, and emphasized the important role of the family in treatment. It enables healthcare professionals to accurately understand the present situation and influencing elements of family resilience in individuals having lung cancer, providing an effective reference and scientific basis for improving the level of family resilience in lung cancer patients in clinical work and developing practical and feasible nursing interventions. Specific interventions include promoting social support networks, encouraging family members to participate in counseling or support groups, and training families on effective communication and coping strategies. These targeted interventions can improve family dynamics and provide better emotional and practical support, ultimately enhancing the overall resilience of both patients and their families during treatment.

## 5 Conclusions

The level of family resilience in individuals with lung cancer is moderate to high, but compared to other cancer patients, the scores are lower, indicating there is still considerable room for improvement. The results of the structural equation model show that cognitive emotional regulation, self‐efficacy, and social support have a notable positive effect on the level of family resilience in patients with lung cancer. Given these findings, healthcare professionals should pay more attention to lung cancer patients at the family level, adopt positive and effective intervention measures, encourage patients to face the disease positively, enhance family communication and intimacy, thereby improving the level of family resilience in lung cancer. Clinically, healthcare professionals can provide training on enhancing self‐efficacy and emotional regulation, and offer support networks to strengthen social support systems. These strategies could not only improve family resilience but also contribute to better psychosocial outcomes for both patients and their families during cancer treatment and care. Furthermore, healthcare policies should incorporate family‐centered care models, ensuring that interventions supporting family resilience are systematically integrated into lung cancer treatment protocols, ultimately enhancing the overall quality of care and support for patients.

## Author Contributions

**Ziyi Shen:** conceptualization (lead), data curation (lead), formal analysis (lead). **Yan Fang:** conceptualization (equal), methodology (equal). **Chengcheng Li:** investigation (equal). **Xin Luo:** investigation (equal). **Junling Cui:** writing – original draft (supporting). **Yanchang Liu:** investigation (supporting). **Jingfang Hong:** project administration (equal), supervision (lead).

## Conflicts of Interest

The authors declare no conflicts of interest.

## Supporting information


Table S1.

## Data Availability

Research data are not shared.

## References

[1] F. Bray , M. Laversanne , H. Sung , et al., “Global Cancer Statistics 2022: GLOBOCAN Estimates of Incidence and Mortality Worldwide for 36 Cancers in 185 Countries,” CA: A Cancer Journal for Clinicians 74, no. 3 (2024): 229–263, 10.3322/caac.21834. 38572751

[2] X. Zhi , J. Shi , and Y. Tian , “Key Points of the White Paper on the Quality of Life of Lung Cancer Patients in China 2022,” Chinese Clinical Journal of Thoracic and Cardiovascular Surgery 30, no. 8 (2023): 1083–1088.

[3] Y. Li , B. Yan , and S. He , “Advances and Challenges in the Treatment of Lung Cancer,” Biomedicine & Pharmacotherapy 169 (2023): 115891, 10.1016/j.biopha.2023.115891. 37979378

[4] H. Brody , “Lung Cancer,” Nature 587, no. 7834 (2020): S7, 10.1038/d41586-020-03152-0. 33208969

[5] H. Zeng , X. Ran , L. An , et al., “Disparities in Stage at Diagnosis for Five Common Cancers in China: A Multicentre, Hospital‐Based, Observational Study,” Lancet Public Health 6, no. 12 (2021): e877–e887, 10.1016/S2468-2667(21)00157-2. 34838194

[6] R. Maguire , L. Lewis , G. Kotronoulas , J. McPhelim , R. Milroy , and J. Cataldo , “Lung Cancer Stigma: A Concept With Consequences for Patients,” Cancer Reports (Hoboken) 2, no. 5 (2019): e1201, 10.1002/cnr2.1201. PMC7941499 32721137

[7] E. J. Morrison , P. J. Novotny , J. A. Sloan , et al., “Emotional Problems, Quality of Life, and Symptom Burden in Patients With Lung Cancer,” Clinical Lung Cancer 18, no. 5 (2017): 497–503, 10.1016/j.cllc.2017.02.008. 28412094 PMC9062944

[8] L. Yan , The Correlation Between Family Resilience and Quality of Life and Self‐Efficacy in Patients With Lung Cancer (Shihezi University, 2021).

[9] Z. Yuji , W. Sijun , and W. F , “Research Progress of Family Resilience,” Occupational and Health 38, no. 14 (2022): 2007–2010.

[10] H. I. McCubbin and J. M. Patterson , “The Family Stress Process: The Double ABCX Model of Adjustment and Adaptation,” Marriage & Family Review 6, no. 1‐2 (1983): 7–37, 10.1300/J002v06n01_02.

[11] X. Jiang , Study of the Effect of Intervention Based on Dual ABC‐X Model on Parental Stress in Children With Chronic Kidney Disease (Zhengzhou University, 2021), 10.27466/d.cnki.gzzdu.2021.005327.

[12] S. Opsomer , E. Lauwerier , J. De Lepeleire , et al., “Resilience in Advanced Cancer Caregiving. A Systematic Review and Meta‐Synthesis,” Palliative Medicine 36, no. 1 (2022): 44–58. 34986698 10.1177/02692163211057749 PMC8796166

[13] M. i. Zhang , Analysis of Family Resilience Status and Influencing Factors of Elderly Lung Cancer Patients (Chinese Medical Sciences University, 2023).

[14] Y. Yang and X. Liao , “Effect of Family Resilience on Negative Mood and Exercise Compliance in Patients After Lung Cancer Surgery,” International Journal of Nursing 39, no. 1 (2020): 31–34.

[15] D. Yan , The Structure of Family Resilience in Middle School Students and its Relationship With Mental Health (Beijing Normal University, 2008).

[16] J. Linjie and D. Yan , “The Relationship Between Traumatic Exposure, Family Resilience and Post‐traumatic growth of Secondary School Students after the Earthquake Disaster,” in Celebration of the 90th Anniversary of Chinese Psychological Society & The 14th National Academic Congress of Psychology, Xi'an, Shaanxi, China (Chinese Psychological Society, 2011).

[17] S. Cohen , T. Kamarck , and R. Mermelstein , “A Global Measure of Perceived Stress,” Journal of Health and Social Behavior 24, no. 4 (1983): 385–396, 10.2307/2136404. 6668417

[18] Y. Zhongting and H. Hanteng , “An Epidemiological Study on the Psychological Stress of Urban Residents During Social Transformatio,” Chinese Journal of Epidemiology 24, no. 9 (2003): 11–15.

[19] H. Hanxue , Relationship Between Social Support, Psychological Congruence and Perceived Stress in Adult Leukemia Patients Undergoing Chemotherapy (North China University of Science and Technology, 2022).

[20] V. Garnefski and S. Ph , “Negative Life Events, Cognitive Emotion Regulation and Emotional Problems,” Personality and Individual Differences 47, no. 4 (2001): 35–50.

[21] Z. Xiongzhao , L. Fusheng , Y. Shujiao , et al., “A Study on the Reliability and Validity of the Chinese Version of Cognitive Emotion Regulation Questionnaire (CERQ‐C),” Chinese Journal of Clinical Psychology 02 (2007): 121–124.

[22] H. Feifel , S. Strack , and V. T. Nagy , “Coping Strategies and Associated Features of Medically Ill Patients,” Psychosomatic Medicine 49, no. 6 (1987): 616–625, 10.1097/00006842-198711000-00007. 3423168

[23] X. Shen and Q. Jiang , “Medical Coping Style Questionnaire Chinese Version of 701 Cases Test Report,” Chinese Behavioral Medicine Science 1 (2000): 22–24.

[24] G. Huang , The Status of Postoperative Body Image and Its Influencing Factors in Patients With Colorectal Cancer (Medical University of Anhui, 2024).

[25] R. P. Y. Y. Bagozzi , “Specification, Evaluation, and Interpretation of Structural Equation Models,” Journal of the Academy of Marketing Science 40, no. 1 (2012): 8–34, 10.1007/s11747-011-0278-x.

[26] K. M. Connor and J. R. Davidson , “Development of a New Resilience Scale: The Connor‐Davidson Resilience Scale (CD‐RISC),” Depression and Anxiety 18, no. 2 (2003): 76–82, 10.1002/da.10113. 12964174

[27] J. X. Zhang and R. Schwarzer , “Measuring Optimistic Self‐Beliefs: A Chinese Adaptation of the General Self‐Efficacy Scale,” Psychologia: An International Journal of Psychology in the Orient 38, no. 3 (1995): 174–181.

[28] Y. L. Yang , L. Liu , X. X. Wang , et al., “Prevalence and Associated Positive Psychological Variables of Depression and Anxiety Among Chinese Cervical Cancer Patients: A Cross‐Sectional Study,” PLoS One 9, no. 4 (2014): e94804. 24722558 10.1371/journal.pone.0094804 PMC3983270

[29] G. S. Pushkarev , G. D. Zimet , V. A. Kuznetsov , and E. I. Yaroslavskaya , “The Multidimensional Scale of Perceived Social Support (MSPSS): Reliability and Validity of Russian Version,” Clinical Gerontologist 43, no. 3 (2020): 331–339, 10.1080/07317115.2018.1558325. 30587089

[30] Q. J. Jiang , “Understand the Social Support Scale,” Chinese Behavioral Medicine Science 1 (2001): 10.

[31] M. Cui , Analysis of the Current Family Resilience of Breast Cancer Patients and Its Influencing Factors (Zunyi Medical University, 2021).

[32] P. Cui , J. Shi , S. Li , M. A. Getu , R. Wang , and C. Chen , “Family Resilience and Its Influencing Factors Among Advanced Cancer Patients and Their Family Caregivers: A Multilevel Modeling Analysis,” BMC Cancer 23, no. 1 (2023): 623, 10.1186/s12885-023-11101-z. 37403053 PMC10320962

[33] Y. Wang , Y. Qiu , L. Ren , H. Jiang , M. Chen , and C. Dong , “Social Support, Family Resilience and Psychological Resilience Among Maintenance Hemodialysis Patients: A Longitudinal Study,” BMC Psychiatry 24, no. 1 (2024): 76, 10.1186/s12888-024-05526-4. 38279114 PMC10811847

[34] S. Lamie , Study on Family Resilience of Lung Cancer Patients and Its Correlation With Hope Level and Understanding Social Support (Guangxi Medical University, 2022).

[35] J. J. Chen , Q. L. Wang , H. P. Li , T. Zhang , S. S. Zhang , and M. K. Zhou , “Family Resilience, Perceived Social Support, and Individual Resilience in Cancer Couples: Analysis Using the Actor‐Partner Interdependence Mediation Model,” European Journal of Oncology Nursing 52 (2021): 101932, 10.1016/j.ejon.2021.101932. 33799020

[36] M. Shao , H. Yang , R. Du , et al., “Family Resilience in Cancer Treatment and Key Influencing Factors: A Systematic Review,” European Journal of Oncology Nursing 66 (2023): 102403. 37690311 10.1016/j.ejon.2023.102403

[37] J. Ke , J. Lin , X. Lin , W. T. Chen , and F. Huang , “Dyadic Effects of Family Resilience on Quality of Life in Patients With Lung Cancer and Spousal Caregivers: The Mediating Role of Dyadic Coping,” European Journal of Oncology Nursing 66 (2023): 102400, 10.1016/j.ejon.2023.102400. 37611499

[38] C. S. Eom , D. W. Shin , S. Y. Kim , et al., “Impact of Perceived Social Support on the Mental Health and Health‐Related Quality of Life in Cancer Patients: Results From a Nationwide, Multicenter Survey in South Korea,” Psychooncology 22, no. 6 (2013): 1283–1290, 10.1002/pon.3133. 22833521

