# Supplementary material for: Family Resilience and Its Influencing Factors Among Patients With Lung Cancer Based on Double ABC‐X Theoretical Framework
Source: Cancer Med. 2025 Apr 15;14(8):e70868. doi: 10.1002/cam4.70868 (PMC11997705; doi:10.1002/cam4.70868)
Supplement: Supplementary file 1 — Table S1. [file CAM4-14-e70868-s001.docx]

**Supplementary Material Table 1:The detailed information regarding the scales**

| Scale Name | Dimension | Number of Items | Score Range | Cronbach's alpha |
| --- | --- | --- | --- | --- |
| Family resilience assessment scale | **Family beliefs** | 17 | 37-82 | 0.912 |
|  | Distress interpretation | 7 | 15-35 |  |
|  | Positive prospecting | 6 | 14-30 |  |
|  | Life excellence | 4 | 6-19 |  |
|  | **Family strengths** | 32 | 53-152 |  |
|  | Problem-solving | 6 | 9-29 |  |
|  | Intimate harmony | 4 | 7-20 |  |
|  | Social support | 4 | 4-20 |  |
|  | Planned life | 3 | 5-15 |  |
|  | Emotional sharing | 4 | 7-20 |  |
|  | Clear communication | 5 | 8-25 |  |
|  | Cooperative coordination | 6 | 9-30 |  |
| Chinese perceived stress scale | Sense of anxiety | 7 | 3-23 | 0.78 |
|  | Sense of loss of control | 7 | 1-24 |  |
| Cognitive emotion regulation questionnaire | **PCER** | 20 | 49-96 | 0.81 |
|  | Acceptance | 4 | 11-19 |  |
|  | Positive Reappraisal | 4 | 8-18 |  |
|  | Reappraisal Planning | 4 | 7-19 |  |
|  | Positive Refocusing | 4 | 7-43 |  |
|  | Rational Analysis | 4 | 4-19 |  |
|  | **NCER** | 16 | 34-65 |  |
|  | Rumination | 4 | 11-19 |  |
|  | Self-Blame | 4 | 4-19 |  |
|  | Catastrophizing | 4 | 5-19 |  |
|  | Blaming Others | 4 | 4-19 |  |
| Medical coping modes questionnaire | Confrontation | 8 | 8-32 | 0.69 |
|  | Avoidance | 7 | 7-28 | 0.60 |
|  | Resignation | 5 | 5-20 | 0.76 |
| Connor davidson resilience scale | Resilience | 13 | 19-50 | 0.910 |
|  | Strength | 8 | 12-32 |  |
|  | Optimism | 4 | 4-16 |  |
| General self-efficacy scale | / | 11 | 11-39 | 0.87 |
| Perceived social support scale | Family support | 4 | 8-28 | 0.899 |
|  | Friend support | 4 | 4-25 |  |
|  | Other support | 4 | 8-28 |  |
